# Delayed Adrenarche may be an Additional Feature of Immunoglobulin Super Family Member 1 Deficiency Syndrome

**DOI:** 10.4274/jcrpe.2512

**Published:** 2016-03-01

**Authors:** Severine Van Hulle, Margarita Craen, Bert Callewaert, Sjoerd Joustra, Wilma Oostdijk, Monique Losekoot, Jan Maarten Wit, Marc Olivier Turgeon, Daniel J. Bernard, Jean De Schepper

**Affiliations:** 1 University Hospital Gent, Department of Pediatrics, Gent, Belgium; 2 University Hospital Gent, Department of Medical Genetics, Gent, Belgium; 3 Leiden University Medical Center, Department of Internal Medicine, Division of Endocrinology, Leiden, Netherlands; 4 Leiden University Medical Center, Department of Pediatrics, Leiden, Netherlands; 5 Leiden University Medical Center, Department of Clinical Genetics, Leiden, Netherlands; 6 McGill University, Department of Pharmacology and Therapeutics, Quebec, Canada

**Keywords:** Immunoglobulin super family member 1 deficiency syndrome, central hypothyroidism, macro-orchidism, delayed adrenarche, Novel mutation

## Abstract

Immunoglobulin super family member 1 (IGSF1) deficiency syndrome is characterized by central hypothyroidism, delayed surge in testosterone during puberty, macro-orchidism, and in some cases, hypoprolactinemia and/or transient growth hormone (GH) deficiency. Our patient was a 19-year-old male adolescent who had been treated since the age of 9 years with GH and thyroxine for an idiopathic combined GH, thyroid-stimulating hormone (TSH), and prolactin (PRL) deficiency. His GH deficiency proved to be transient, but deficiencies of TSH and PRL persisted, and he had developed macro-orchidism since the end of puberty. Brain magnetic resonance imaging and PROP1 and POU1F1 sequencing were normal. A disharmonious puberty (delayed genital and pubic hair development, bone maturation, and pubertal growth spurt, despite normal testicular growth) was observed as well as a delayed adrenarche, as reflected by very low dehydroepiandrosterone sulfate and delayed pubarche. Direct sequencing of the IGSF1 gene revealed a novel hemizygous mutation, c.3127T>C, p.Cys1043Arg. Pathogenicity of the mutation was demonstrated in vitro. Male children with an idiopathic combined GH, PRL, and TSH deficiency, showing persistent central hypothyroidism but transient GH deficiency upon retesting at adult height, should be screened for mutations in the IGSF1 gene, especially when macro-orchidism and/or hypoprolactinemia are present. We suspect that delayed adrenarche, as a consequence of PRL deficiency, might be part of the clinical phenotype of patients with IGSF1 deficiency.

**WHAT IS ALREADY KNOWN ON THIS TOPIC?**

Loss of function of the immunoglobulin super family member I (IGSF1) gene is characterized in males by central hypothyroidism, delayed testosterone rise in puberty despite normal timing of testicular enlargement, and adult macro-orchidism. Approximately 15% of male patients have transient growth hormone deficiency and 65% have hypoprolactinemia. Normally, the mature glycoform of IGSF1 is localized at the cell surface, and most loss-of-function mutations impair its glycosylation or trafficking to the cell membrane.

**WHAT THIS STUDY ADDS?**

In addition to a delayed pubertal surge in testosterone, we documented a delayed increase in dehydroepiandrosterone sulfate and delayed pubarche in our patient. We suspect that a delayed adrenarche might be part of the clinical phenotype of patients with IGSF1 deficiency and contributes to delayed bone maturation.

## INTRODUCTION

Loss-of-function of the immunoglobulin super family member 1 gene (IGSF1, OMIM#300888) causes an X-linked syndrome, characterized in males by congenital central hypothyroidism, delayed testosterone rise in puberty despite normal timing of testicular enlargement, adult macro-orchidism, and in some cases deficiencies of prolactin (PRL) and/or growth hormone (GH) (1,2,3). A small proportion of heterozygous females show central hypothyroidism, PRL deficiency, and/or delayed menarche (3). Human IGSF1 messenger ribonucleic acid is abundantly expressed in the adult and developing anterior pituitary gland and testis. The gene encodes a plasma membrane glycoprotein (1).

With 31 patients described to date, the variability of the phenotype may not yet be fully characterized (1,4,5). In the present case, IGSF1 deficiency was diagnosed at the age of 19 years, based on a history of transient GH deficiency, persistent central hypothyroidism, and hypoprolactinemia, and the finding of macro-orchidism. The close follow-up during GH replacement, which was started one year before the onset of puberty, permitted a detailed recording of the genital and pubic hair development, pubertal growth spurt, and bone maturation. Besides a delayed pubertal surge in testosterone, a delayed increase in dehydroepiandrosterone sulfate (DHEAS) was documented in our patient. We suspect that delayed adrenarche might be part of the clinical phenotype of patients with IGSF1 deficiency.

## CASE REPORT

The boy was first seen at the pediatric endocrinology clinic at the age of 9 years for reduced growth velocity since the age of 3 years. He was born after 39 weeks of gestation, with a birth weight of 3750 grams and a birth length of 52 cm. A slightly prolonged neonatal jaundice was noted. His neurocognitive development was normal. From 3 years on, he intermittently received standard corticoid inhalation therapy for allergic asthma and corticoid ointments for chronic eczema.

**Figure 1:**
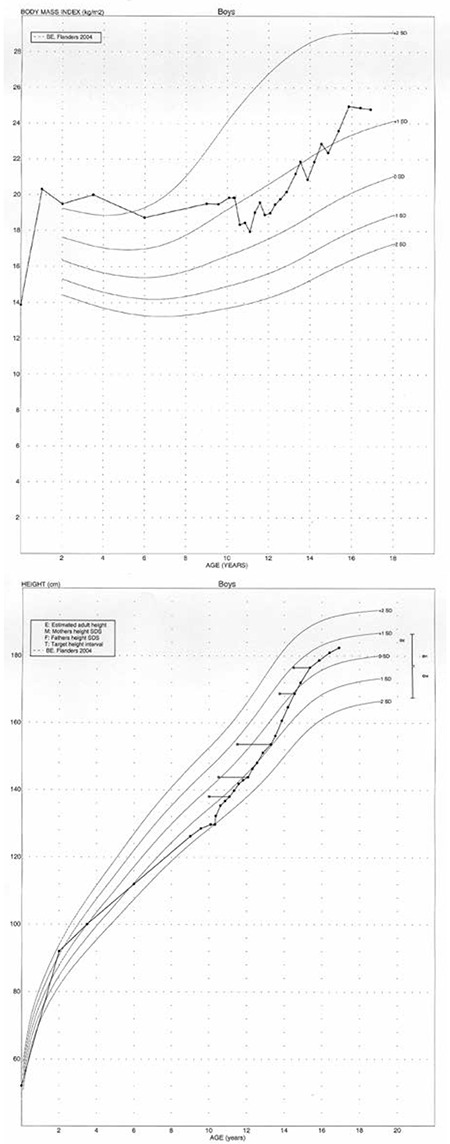
Height, bone age, and body mass index data. Levothyroxine treatment was initiated at age 9 years and 3 months. At age 10 years and 4 months, growth hormone replacement therapy was started

His initial work-up showed a delayed bone age (6.25 years at a chronological age of 9.1 years) and a low IGF-1 concentration of 72 ng/mL (reference range for age: 74-388 ng/mL). Serum thyroid-stimulating hormone (TSH) was normal (1.6 µU/mL, reference range 0.28-4.3 µU/mL), free thyroxine (fT_4_) was on the lower limit of the reference range (0.9 ng/dL, reference range 0.9-1.7 ng/dL), and free triiodothyronine (fT_3_) was normal (3.4 ng/dL, reference range 2.57-4.43 ng/dL). Serum PRL level was unmeasurable (<0.5 ng/mL), while basal cortisol was normal (15 µg/dL). He was treated with levothyroxine, inducing a temporary catch-up growth (Figure 1). After 6 months, partial GH deficiency was suspected based on a GH peak of 9.4 ng/mL after glucagon stimulation. The low GH reserve was confirmed at insulin tolerance testing after priming with testosterone (peak GH 6.6 ng/mL). Brain magnetic resonance imaging, including the hypothalamic-pituitary region, was normal. Because of the combination of central hypothyroidism, GH deficiency, low PRL status, and normal pituitary imaging, genetic testing of PROP1 and POU1F1 was performed, but no mutations were found.

Based on these findings, he was diagnosed with idiopathic combined GH, TSH, and PRL deficiency. GH replacement therapy (0.03 mg/kg/day) was initiated at the age of 10 years and 4 months, resulting in rapid catch-up growth. Follow-up examinations (Table 1) revealed a disharmonious puberty with delayed genital and pubic hair development and testosterone surge, but normal timing of the increase in testicular size. Excessive testicular growth became evident at the end of puberty (Table 1). DHEAS measurements were repeatedly low. Bone maturation progressed slowly. Low dose adrenocorticotropic hormone (ACTH) testing at 14 years and 7 months old showed a normal cortisol increase (serum cortisol 20.3 µg/dL at 30 minutes). PRL levels remained undetectable (<0.5 ng/mL) throughout the whole follow-up.

Z-scores for height increased during pubertal development, in accordance with increasing serum IGF-1 concentrations, while fT^4^ concentrations remained normal during treatment with levothyroxine. Pubertal growth slowed down around the age of 16.5 years (height increase <3 cm/year). After stopping GH treatment for 3 months, combined insulin-thyrotropin-releasing hormone (TRH)-gonadotropin-releasing hormone testing showed a low normal TSH (peak value: 6.3 mU/L) and very low PRL reserve (peak value: 5.8 ng/L), but a normal GH (peak value 15.3 ng/mL), cortisol, follicle stimulating hormone (FSH) and luteinizing hormone (LH) response. Basal serum FSH (6.6 U/L) was higher than LH (3.0 U/L), although both were within the reference ranges and testosterone was normal (512.1 ng/dL). Basal fT^4^ concentration was low (0.8 ng/dL) due to poor compliance in the last month. At his last visit, at the age of 19 years, his height was 182.8 cm (0.5 standard deviation score [SDS]) and his weight 89.3 kg (body mass index of 27 kg/m2, 1.5 SDS). His orchidometric testicular volume was >30 mL bilaterally, and his pubertal status A3P5G5. Total pubertal height gain was 41 cm.

The combination of persistent central hypothyroidism, transient partial GH deficiency, and macro-orchidism led to the suspicion of IGSF1 deficiency (1). The patient gave his informed consent for IGSF1 gene analysis and publication of his clinical history. His mother declined carrier testing.

Mutation analysis of the IGSF1 gene was performed by direct sequencing. The glycosylation and expression at the plasma membrane was examined in heterologous HEK293 cells, as described in (1). To assess plasma membrane trafficking, HEK293 cells were transfected with pcDNA3.0 (empty vector), or with expression vectors for C-terminal HA-tagged forms of the wild-type (IGSF1-HA wt) or mutant IGSF1 (IGSF1-HA C1043R; c.3127T>C, p.Cys1043Arg). Plasma membrane proteins were biotinylated prior to cell lysis. IGSF1 protein was immunoprecipitated (IP) with an HA antibody and then resolved by SDS-PAGE under reducing conditions. Biotinylated IGSF1 at the plasma membrane was detected by streptavidin-horseradish peroxidase. Efficacy of the IP was assessed by HA immunoblot.

**Figure 2:**
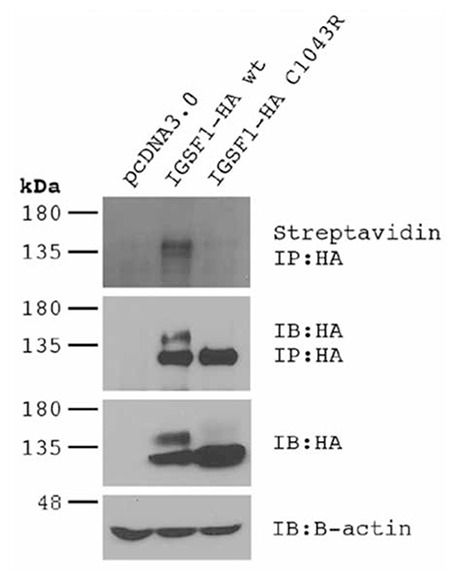
Results of immunoblotting assays. Biotinylated Immunoglobulin super family member 1 (IGSF1) at the plasma membrane was detected by streptavidin-horseradish peroxidase (top panel). Note the appearance of a band exclusively in the wild-type lane. Efficacy of the immunoprecipitated was assessed by HA immunoblot (IB; second panel from top). Note the appearance of a doublet in the wild-type lane and a single band in the mutant IGSF1 lane, indicating that the latter fails to acquire mature carbohydrates. The same banding pattern was observed when the proteins were analyzed by direct immunoblotting (third panel from top). The bottom panel confirmed equal loading of the samples in the HA:IB panel

As observed previously for other pathogenic missense mutations, IGSF1 harboring the Cys1043Arg mutation does not acquire mature glycosylation and fails to traffic from the endoplasmic reticulum to the plasma membrane (Figure 2).

## DISCUSSION

We described the first Belgian patient with a novel IGSF1 mutation and presented detailed longitudinal data on the patient’s growth, pubertal development as well as his testicular and adrenal functions. The main characteristics of this newly described genetic syndrome are congenital hypothyroidism of central origin and macro-orchidism. The diagnosis of central hypothyroidism is rarely made at birth as most neonatal screening programs for congenital hypothyroidism are based solely on the measurement of TSH. In the Netherlands, where neonatal screening of both thyroxine and TSH levels allows for an early diagnosis of central hypothyroidism, the incidence of IGSF1 deficiency is estimated at approximately 1:100 000 (2). Slow linear growth and increased adiposity can be the presenting sign of central hypothyroidism during childhood, as observed in our case. In the reported cases, the hypothyroidism was mild, with a mean serum fT_4_ level at the lower limit of the reference range (1,3). TRH stimulation testing in our patient in young adulthood showed a weak TSH response, as has been reported in most cases, although delayed and exaggerated responses have also been observed (1,5).

In a small proportion (15%) of patients with the IGSF1 deficiency syndrome, a partial and transient GH deficiency also contributes to the growth delay. Our case showed a decreased GH reserve despite correction of his hypothyroid state and priming with testosterone, and his GH reserve normalized only after reaching young adulthood. This observation is in line with the initial report on four patients with transient partial GH deficiency (1). Based on murine expression of IGSF1 protein in thyrotropes, somatotropes, and lactotropes in the pituitary gland, a role for IGSF1 in pituitary GH production and/or secretion appears likely.

We recorded by regular examinations a disharmonious pubertal development (normal timing of testicular growth, but a delayed surge of serum testosterone) in our patient. In patients with the IGSF1 deficiency syndrome, prepubertal testicular size is usually normal and testicular enlargement starts at a normal age. However, testicular volumes exceeds the reference range during puberty and enlargement may continue further in adulthood (2). Testicular size is determined by Sertoli cell number and is mainly dependent on FSH and thyroid hormone levels (6). In reported cases of boys with IGSF1 deficiency, serum FSH concentrations were always higher than serum LH values, although still within the normal range. As a consequence of the delayed testosterone production, pubic hair development and the pubertal growth spurt are delayed in IGSF1 deficiency. Nevertheless, normal testosterone levels are observed in young adulthood (1).

Interestingly, in our case we also observed delayed adrenarche, as reflected by DHEAS levels below the reference range and the absence of pubarche until the age of 13.8 years. Although delayed pubic hair development has been observed previously, no data on adrenal androgens have been reported in patients with IGSF1 deficiency. However, delayed adrenarche has also been observed in boys with mutations in the PIT1/POU1F1 gene causing GH, TSH, and PRL deficiency and therefore resembling IGSF1 deficiency (7). It is unlikely that GH or TSH deficiency are responsible for the delayed adrenarche, since our patient received replacement therapy for both deficiencies. Furthermore, patients with isolated GH deficiency are known to have normal adrenal androgen levels (8) and the expression of the TSH receptor in the adrenal cortex is very low (9). We suspect that the delayed adrenarche in both IGSF1 and POU1F1 defects might be caused by the low PRL secretion. PRL receptors are highly expressed in the adrenal cortex and synergize with ACTH to augment secretion of adrenal androgens (10,11,12). Also, inducing decreased PRL by exogenous dopamine reduces DHEAS levels, whereas hyperprolactinemia is associated with elevated DHEAS (13,14). The low DHEAS production might contribute to the delayed pubic hair growth, but also to the delayed bone maturation, which was observed before as well as during GH therapy.

Up to now, systematic IGSF1 mutation analysis has not been performed in larger cohorts of patients with transient or persistent GH deficiency in combination with central hypothyroidism and low PRL levels. Most institutions start with analysis of the PROP1 gene in cases of combined pituitary hormone deficiency. However, molecular changes in many patients currently remain unexplored (15). More comprehensive and faster genetic screening techniques will gain importance in the diagnosis and management of these pituitary hormone deficiencies and will detect family members at risk. IGSF1 deficiency is inherited in an X-linked pattern with reduced penetrance in females. However, since the deficiency may manifest itself as central hypothyroidism, hypoprolactinemia, and delayed menarche in female carriers, mutation analysis in at risk family members is recommended.

Male children with an idiopathic combined GH and TSH deficiency, showing a persistent central hypothyroidism but a transient GH deficiency, should be screened for loss-of-function mutations or deletions of the IGSF1 gene, especially when delayed puberty and macro-orchidism are present. IGSF1 deficiency may be associated with delayed adrenarche, possibly caused by PRL deficiency.

## Ethics

Informed Consent: obtained.

Peer-review: External and Internal peer-reviewed.

## Figures and Tables

**Table 1:**
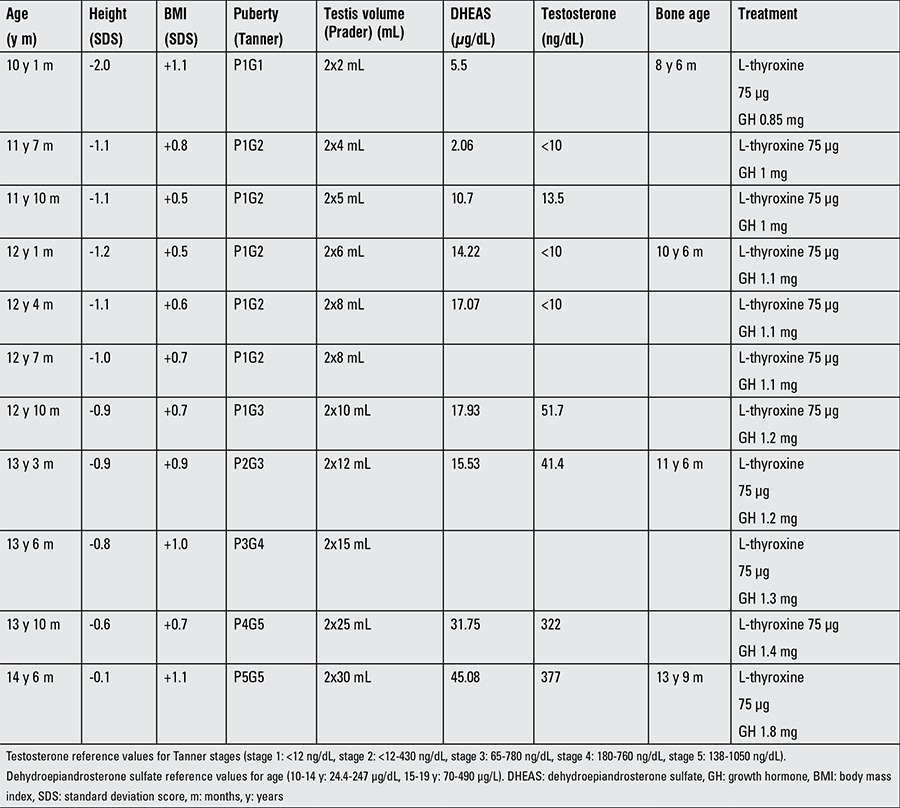
Auxological and hormonal data during adolescence

## References

[1] Sun Y, Bak B, Schoenmakers N, Trotsenburg AS, Oostdijk W, Voshol P, Cambridge E, White JK, le Tissier P, Gharavy SN, Martinez-Barbera JP, Stokvis-Brantsma WH, Vulsma T, Kempers MJ, Persani L, Campi I, Bonomi M, Beck-Peccoz P, Zhu H, Davis TM, Hokken-Koelega AC, Del Blanco DG, Rangasami JJ, Ruivenkamp CA, Laros JF, Kriek M, Kant SG, Bosch CA, Biermasz NR, Appelman-Dijkstra NM, Corssmit EP, Hovens GC, Pereira AM, den Dunnen JT, Wade MG, Breuning MH, Hennekam RC, Chatterjee K, Dattani MT, Wit JM, Bernard DJ (2012). Loss-of-function mutations in IGSF1 cause an X-linked syndrome of central hypothyroidism and testicular enlargement. Nat Genet.

[2] (2013;1:e24883). Joustra SD, van Trotsenburg AS, Sun Y, Losekoot M, Bernard DJ, Biermasz NR, Oostdijk W, Wit JM. IGSF1 deficiency syndrome: A newly uncovered endocrinopathy.

[3] Joustra SD, Schoenmakers N, Persani L, Campi I, Bonomi M, Radetti G, Beck-Peccoz P, Zhu H, Davis TM, Sun Y, Corssmit EP, Appelman-Dijkstra NM, Heinen CA, Pereira AM, Varewijck AJ, Janssen JA, Endert E, Hennekam RC, Lombardi MP, Mannens MM, Bak B, Bernard DJ, Breuning MH, Chatterjee K, Dattani MT, Oostdijk W, Biermasz NR, Wit JM, Trotsenburg AS (2013). The IGSF1 deficiency syndrome: characteristics of male and female patients. J Clin Endocrinol Metab.

[4] Tajima T, Nakamura A, Ishizu K (2013). A novel mutation of IGSF1 in a Japanese patient of congenital central hypothyroidism without macroorchidism. Endocr J.

[5] Nakamura A, Bak B, Silander TL, Lam J, Hotsubo T, Yorifuji T, Ishizu K, Bernard D, Tajima T (2013). Three novel IGSF1 mutations in four Japanese patients with X-linked congenital central hypothyroidism. J Clin Endocrin Metab.

[6] Wagner MS, Wajner SM, Maia AL (2008). The role of thyroid hormone in testicular development and function. J Endocrinol.

[7] Taha D, Mullis PE, Ibanez L, Zegher F (2005). Absent or delayed adrenarche in Pit-1/POU1F1 deficiency. Horm Res.

[8] Ilondo MM, Vanderschueren-Lodeweyckx M, Vlietinck R, Pizarro M, Malvaux P, Eggermont E, Eeckels R (1982). Plasma Androgens in children and adolescent Part II. A longitudinal study in patients with hypopituitarism. Horm Res.

[9] Dutton CM, Joba W, Spitzweg C, Heufelder AE, Bahn RS (1997). Thyrotropin receptor expression in adrenal, kidney, and thymus. Thyroid.

[10] Glasow A, Breidert M, Haidan A, Anderegg U, Kelly PA, Bornstein SR (1996). Functional aspects of the effect of prolactin [PRL] on adrenal steroidogenesis and distribution of the PRL receptor in the human adrenal gland. J Clin Endocrinol Metab.

[11] Higuchi K, Nawata H, Maki T, Higashizima M, Kato K, Ibayashi H (1984). Prolactin has a direct effect on adrenal androgen secretion. J Clin Endocrinol Metab.

[12] Bole-Feysot C, Goffin V, Edery M, Binart N, Kelly PA (1998). Prolactin [PRL] and its receptor: actions, signal transduction pathways and phenotypes observed in PRL receptor knockout mice. Endocr Rev.

[13] De Zegher F, Wouters P, Schetz M, Verwaest C, Ferdinande P, Lauwers P (1995). Dehydroepiandrosterone sulphate in critical illness: effect of dopamine. Clin Endocrinol [Oxf].

[14] Schiebinger RJ, Chrousos GP, Cutler GB, Loriaux DL (1986). The effect of serum prolactin on plasma adrenal androgens and the production and metabolic clearance rate of dehydroepiandrosterone sulfate in normal and hyperprolactinemic subjects. J Clin Endocrinol Metab.

[15] Reynaud R, Gueydan M, Saveanu A, Vallette-Kasic S, Enjalbert A, Brue T, Barlier A (2006). Genetic screening of combined pituitary hormone deficiency: experience in 195 patients. J Clin Endocrinol Metab.

